# Psychological and social factors influencing mother–child bonding in the first year after birth: a model for promoting infant and maternal well-being

**DOI:** 10.3389/fpsyg.2025.1588433

**Published:** 2025-04-30

**Authors:** Valentina Lucia La Rosa, Dario Alparone, Elena Commodari

**Affiliations:** ^1^Department of Educational Sciences, University of Catania, Catania, Italy; ^2^Department of Psychology, Faculty of Letters and Human Sciences, University of Western Brittany, Brest, France

**Keywords:** bonding, postpartum, child development, maternal mental health, social support

## Abstract

**Introduction:**

This study aimed to examine how maternal mental health, sleep quality, and social support influence mother-infant bonding during the first year after childbirth.

**Methods:**

A total of 1,495 mothers participated by completing standardized questionnaires that assessed the quality of bonding with their infants, experiences of childbirth-related post-traumatic stress, symptoms of postpartum depression, sleep disturbances, and levels of social support.

**Results:**

The results indicated that 49.6% of mothers experienced childbirth-related post-traumatic stress, 32.2% reported symptoms of postpartum depression, and 50.7% experienced sleep disturbances. Regression analysis showed that postpartum depression, poor sleep quality, older maternal age, emergency cesarean delivery, and low levels of social and partner support hurt the quality of the mother-infant bond. Depressive symptoms emerged as the strongest negative predictor, whereas adequate social and partner support positively influenced bonding.

**Discussion:**

These findings highlight the need for early screening and interventions aimed at improving maternal mental health and social support to promote positive mother-infant bonding and healthy child development.

## 1 Introduction

The relationship between a mother and her infant is essential for early childhood development (Bowlby, 1969; Ainsworth, 1978), as mothers typically serve as primary caregivers in the postpartum period. This unique role is shaped by biological factors, including physical bonding during pregnancy and breastfeeding and the caregiving duties that mothers typically take on. As the primary attachment figure, mothers significantly influence their children’s emotional security and overall growth (Sullivan et al., 2011). Although other caregivers, such as fathers or extended family members, can also play a role in attachment, the mother’s influence is especially crucial during the initial months of the child’s life (Bowlby, 1988).

The emotional connection that develops between mother and infant after birth is known as bonding, and it ensures the protection and nurturing of the newborn (Klaus and Kennell, 1982; Nonnenmacher et al., 2016). Specifically, bonding refers to the maternal component of the reciprocal emotional connection between the primary caregiver and the child, which begins during pregnancy and gradually strengthens during the early stages of childhood (Davies et al., 2021; Fallon et al., 2021). Positive bonding fosters secure attachment, giving infants a sense of security and allowing them to confidently explore their environment and form healthy relationships later in life (Cassidy et al., 2013; Holmes, 2014).

Establishing secure attachment during the postpartum period is critical for a child’s future development, as the quality of this early bond can have significant and lasting effects on mental health and overall well-being. Children who develop secure attachments show better emotional regulation, stronger social connections, and improved stress management skills (Waters et al., 2010; Tang et al., 2023). They tend to be more resilient, less susceptible to anxiety and depression, and achieve better academic performance (Bergin and Bergin, 2009; Boldt et al., 2016; Khan et al., 2020). Furthermore, secure attachment promotes a positive self-image and improves the ability to build healthy relationships throughout life (Martin Quintana et al., 2023). It is also linked to physiological benefits, such as enhanced immune function and reduced stress hormone levels, contributing to overall health and resilience (Cassidy et al., 2013; Pietromonaco and Powers, 2015). The positive effects of secure attachment extend into adolescence and adulthood and influence career success, relationship satisfaction, and overall life satisfaction (Sperling and Berman, 1994; Çuhadaroğlu Çetin et al., 2010; Sagone et al., 2023). Therefore, promoting a healthy mother–child relationship from the earliest months of life is critical for ensuring proper development later in life.

Several factors can hinder the development of a strong mother–child bond during the postpartum period. Maternal mental health issues such as postpartum depression are significant barriers (Santona et al., 2015; Sliwerski et al., 2020; Li, 2023). These conditions can impair a mother’s ability to engage in the sensitive and responsive caregiving necessary for positive bonding and secure attachment and are associated with adverse maternal perceptions of the attachment relationship (Toth et al., 2009; De Falco et al., 2014; McCabe, 2014). In this regard, traumatic birth experiences and childbirth-related post-traumatic stress disorder (PTSD) can significantly impair mothers’ mental health (Ayers et al., 2006; Kjerulff et al., 2021). Maternal experiences of trauma during childbirth can lead to various psychological issues, such as feelings of detachment or hypervigilance, which may impede the formation of a secure bond between the mother and infant. Additionally, studies have found a link between specific delivery methods, such as instrumental delivery or emergency cesarean section, and a higher risk of developing postpartum PTSD, along with bonding challenges (Kjerulff et al., 2021; Sommerlad et al., 2021; La Rosa et al., 2024).

Sleep disturbances are associated with a significant risk to the mother-infant bond, with implications for the health of both. In the postpartum period, parents often experience disrupted sleep because their infants wake up frequently at night (Gay et al., 2004). Infants typically wake up at least once per night (Hysing et al., 2014), which increases alertness for parents who must care for and feed them (Hunter et al., 2009). Although the total sleep duration may remain constant, mothers’ sleep continuity significantly decreases, particularly in the immediate postpartum period (Montgomery-Downs et al., 2010). However, prolonged sleep disturbances can persist, as 20–30% of infants and toddlers wake at night beyond the early postpartum period (King et al., 2020). Insomnia and poor sleep quality during the postpartum period are associated with higher levels of stress, depression, and anxiety (Baglioni et al., 2022; Palagini et al., 2023), reducing the mother’s emotional availability and responsiveness, which is crucial for a positive bond with her infant (Hairston et al., 2016). Studies have shown that mothers experiencing sleep disturbances may exhibit less sensitive caregiving behaviors, negatively impacting their interactions with their babies (Tikotzky et al., 2010; Tikotzky et al., 2012; Tikotzky, 2016; King et al., 2020). When a mother is sleep-deprived, her ability to cope with stress and regulate emotions diminishes, often leading to increased irritability, fatigue, and depressive symptoms (Kendall-Tackett, 2007; Goyal et al., 2009).

In addition, factors such as stress and fatigue associated with caring for newborns, lack of social support, and socioeconomic challenges may play a significant role (Saharoy et al., 2023). Issues related to physical health and complications during childbirth can also hinder the bonding process (Smorti et al., 2020; Sommerlad et al., 2021; Oddo-Sommerfeld et al., 2022). Moreover, stressful life events, including conflicts within a couple and financial struggles, can adversely affect a mother’s mental health and ability to bond with her child (Hutchens and Kearney, 2020; Della Corte et al., 2022). Societal expectations and pressures regarding motherhood can further complicate these challenges and discourage mothers from seeking assistance (De Sousa Machado et al., 2020). Therefore, it is crucial to establish a comprehensive support network for mothers that addresses emotional and practical needs during pregnancy and the postpartum period (De Sousa Machado et al., 2020; Al-Mutawtah et al., 2023).

While risk factors present a considerable challenge, protective factors - especially support from partner and broader social networks - are crucial in promoting a positive bond between mother and infant. The presence of a supportive partner is one of the most important influences on a mother’s emotional resilience and ability to nurture.

Indeed, partner support has been shown to play a critical role in alleviating maternal stress and enhancing the bond between mothers and infants. The emotional availability of the partner, active participation in caregiving, and hands-on assistance with infant-related tasks resulted in a substantial reduction in maternal burden and an improvement in the mother’s capacity to engage in sensitive caregiving behaviors. Research findings suggest that mothers with supportive partners exhibit higher levels of emotional readiness, reduced parenting stress, and enhanced caregiver satisfaction (Cheng et al., 2016; Almutairi et al., 2017). The quality of the couple’s relationship has been demonstrated to impact maternal mental health directly, with positive communication and shared responsibilities correlating with reduced risks of depression and anxiety (Hutchens and Kearney, 2020; La Rosa et al., 2024).

The COVID-19 pandemic highlights the crucial role of partner support. Studies indicate that the presence of the partner during childbirth significantly supported the mental health of mothers at a time of increased stress and uncertainty. Mothers who had their partner by their side experienced lower levels of anxiety, depression, and post-traumatic stress symptoms than those who gave birth alone due to hospital restrictions (Oddo-Sommerfeld et al., 2022). A supportive partner during childbirth offers emotional stability and helps lessen the psychological effects of pandemic-related challenges (La Rosa et al., 2024). Conversely, the absence of the partner during this critical time was linked to increased psychological distress, underscoring the importance of partner involvement in maternal well-being during crises (Oddo-Sommerfeld et al., 2022; La Rosa et al., 2024). Furthermore, ongoing partner support in the postpartum period enhances maternal mental health by creating a nurturing environment that promotes positive bonding (Modak et al., 2023). The key elements of this support include emotional availability, shared responsibilities, and practical help in caring for the newborn (Antoniou et al., 2022).

The partner’s role is crucial, but a wider social network also offers significant emotional and practical support. Assistance from family, friends, and community resources can alleviate the impact of stressors, such as sleep deprivation and financial strain. Emotional support enhances mothers’ confidence and overall well-being, whereas practical help with chores and childcare enables mothers to concentrate on forming strong bonds with their children (Al-Mutawtah et al., 2023).

## 2 Study aims

The main aim of this study was to identify the risk and protective factors that affect the development of positive mother-infant bonding during the first year of the child’s life. This study specifically examined how birth trauma, postpartum depression, sleep issues, perceived social support, and relationship satisfaction with partner played a role in mother-infant bonding.

The specific objectives of this study were as follows:

1. To create a predictive model for mother-infant bonding during the postpartum period that considers maternal mental health, birth experiences, sleep quality, social support, and infant age.
2. To investigate how traumatic birth experiences, symptoms of postpartum depression, and maternal sleep disturbances negatively affect mother-infant bonding.
3. To assess the role of perceived social support and partner relationship satisfaction in promoting positive bonding between mothers and infants.
4. To evaluate how these variables differ by infant age and to understand how risk and protective factors vary at different stages of a child’s first year.

In conclusion, this study aimed to provide a better understanding of the factors that influence early attachment and guide interventions that encourage positive bonding outcomes among diverse groups of mothers.

## 3 Materials and methods

### 3.1 Study design and participants

This cross-sectional observational study was part of a larger project investigating the psychological aspects of postpartum bonding among Italian mothers. Participants were eligible for inclusion if they met the following criteria: being 18 years of age or older, having given birth to a child aged 0–12 months at the time of participation, having sufficient knowledge of the Italian language, and providing valid informed consent. Women were excluded from the study if they (a) were younger than 18 years of age, (b) had a diagnosed psychiatric disorder and/or were undergoing psychopharmacological treatment, (c) did not have sufficient understanding of the Italian language, or had verbal communication limitations that prevented completion of the research protocol, or (d) had a child with medical complications or a diagnosed neurodevelopmental disorder.

Participants completed a survey distributed through social media platforms and forums dedicated to motherhood, parenting, and perinatal well-being. The survey was available from December 2022 to December 2023. Participation was voluntary and anonymous, and no compensation was offered. Measures were taken to ensure data quality, including preventing multiple submissions from the same IP address and a manual review of responses to identify and exclude duplicates based on demographic and response pattern inconsistencies. Before participation, detailed information about the study objectives, procedures, and expected duration was provided, and informed consent was obtained electronically.

The research complied with the ethical standards outlined in the Declaration of Helsinki, the Code of Ethics of Italian Psychologists (L. 18.02.1989, n. 56), the Italian Data Protection Law (DLGS 196/2003), and the Code of Ethics for Psychological Research (March 27, 2015), as approved by the Italian Association of Psychologists. The study protocol was approved by the Internal Ethics Review Board of the Department of Educational Sciences of the University of Catania (prot. n. Ierb-Edunict-2023.05.23/04).

### 3.2 Measures

The online survey began with a section where participants shared detailed sociodemographic information, including their age, education level, marital status, and employment status. They were also asked about their childbirth experiences, such as the type of delivery (vaginal or cesarean), any pregnancy risk factors, whether they were hospitalized during pregnancy, previous miscarriages or preterm births, and birth weight and health status of their newborns.

The second section featured a thorough set of questions regarding perceived social support and satisfaction with relationships with partners. Finally, the participants completed a series of standardized questionnaires designed to evaluate mother–child bonding, experiences of trauma related to childbirth, symptoms of postpartum depression, and issues with sleep disturbances.

The standardized questionnaires used in this study are described below.

#### 3.2.1 Maternal Postnatal Attachment Scale (MPAS)

The MPAS assesses the strength of the mother-infant bond. The MPAS consists of 19 self-report items and is designed for mothers with infants up to 1 year of age. Sample items include statements about feelings of irritation during caregiving, anxiety when with the baby, and pride when others are present. Responses are scored on a five-point scale, ranging from 1 to 5. The total score is calculated by adding up the scores of all items, with a possible range of 19 to 95. A higher score indicates a stronger mother-infant bond, while a lower score suggests a weaker bond. The MPAS showed good reliability in this study, with a Cronbach’s *α* of 0.78.

#### 3.2.2 Impact of Event Scale-Revised (IES-R)

The IES-R evaluates distress associated with traumatic experiences (Creamer et al., 2003). Consisting of 22 items rated on a 5-point Likert scale from 0 (“not at all”) to 4 (“extremely”), this questionnaire provides an overall post-traumatic stress score and subscores for the three primary aspects of post-traumatic stress: intrusion, avoidance, and hyperarousal. A score of 24 or higher indicates significant post-traumatic stress symptoms. This study used the Italian version of the scale (Craparo et al., 2013), with items adapted to focus on childbirth-related experiences (e.g., “memories brought back feelings about childbirth”). The scale showed excellent internal consistency in this study, with an overall reliability coefficient of *α* = 0.90.

#### 3.2.3 Edinburgh Postnatal Depression Scale (EPDS)

The EPDS, developed by Cox et al. (1987), is a commonly used instrument for identifying symptoms of postpartum depression. This assessment tool consists of 10 items, such as “I have been able to laugh and see the funny side of things,” “I have been so unhappy that I have had trouble sleeping,” and “I have been so unhappy that I have been crying.” Participants rate each statement on a four-point scale, reflecting their emotional state over the past week. According to a recent study by Orsolini et al. (2022), a cumulative score of 12 or higher indicates the need for additional assessment of depressive symptoms in the postpartum period. This study showed that the EPDS has high reliability, with a Cronbach’s *α* of 0.86.

#### 3.2.4 Pittsburgh Sleep Quality Index (PSQI)

The PSQI (Buysse et al., 1989) is a self-administered 18-item questionnaire that assesses sleep patterns and disturbances over the past month. It includes seven components: perceived sleep quality, time to fall asleep, sleep duration, sleep efficiency, sleep disturbances, use of sleep medications, and impairment of daytime functioning. Each component is scored on a scale of 0 to 3, with 0 indicating no problems and 3 indicating severe problems. The total score is calculated by summing the individual component scores, ranging from 0 to 21. Higher scores suggest poorer sleep quality. A score exceeding 5 indicates significant sleep disturbance, characterized by notable issues in at least two components or moderate difficulties in three or more areas. This threshold effectively discriminates between individuals with good and poor sleep quality. In the study sample, the questionnaire demonstrated strong internal consistency (Cronbach’s *α* = 0.83).

#### 3.2.5 Maternity Social Support Scale (MSSS)

The MSSS (Webster et al., 2000) is a self-report instrument of 6 items designed to evaluate social support during pregnancy and after childbirth. Participants respond to statements such as “My family is always there for me” and “My husband/partner helps me a lot,” using a five-point Likert scale ranging from 1 (never) to 5 (always). The interpretation of scores is as follows: below 18 indicates insufficient social support, 19 to 24 suggests moderate social support, and above 24 signifies adequate social support. This study used the Italian-validated version of the scale (Dabrassi et al., 2009). In the study sample, the MSSS demonstrated reliability slightly under the 0.70 threshold (Cronbach’s α = 0.67). Nevertheless, this value can be deemed acceptable considering the limited number of items in the questionnaire.

### 3.3 Statistical analyses

To determine the minimum required sample size for this study, a preliminary power analysis was performed using G*Power version 3.1.9.6 (Faul et al., 2007). To evaluate each scale’s reliability, the items’ internal consistency was measured using Cronbach’s alpha. A threshold of α > 0.70 was established as the minimum acceptable level for reliability (DeVellis and Thorpe, 2022).

For continuous variables, means (M) and standard deviations (SD) are utilized as descriptive measures. In contrast, categorical variables are characterized by their frequencies and percentages.

Differences in questionnaire scores by age of the child, divided into three trimesters (0–4 months, 5–8 months, and 9–12 months), were analyzed using multivariate analysis of variance (MANOVA). This method was chosen because it allows for the simultaneous examination of multiple dependent variables. It is particularly useful when assessing the interaction between related outcomes, such as scores on different questionnaires. The MANOVA also controls for the potential inflation of Type I errors that can occur with multiple ANOVA tests when multiple dependent variables are examined (Finch, 2016). Pillai’s trace was used to assess model significance, and effect sizes were calculated using partial η^2^, with values of 0.01, 0.06, and 0.14, representing small, medium, and large effects, respectively (Cohen, 1988). *Post hoc* comparisons were performed using Bonferroni adjustments to identify specific group differences. Before performing the MANOVA, the assumptions of multivariate normality, homogeneity of variance–covariance matrices, and absence of multicollinearity were checked.

Linear multiple regression analysis was used to identify predictors of mother-infant bonding in the sample. The MPAS score was used as the dependent variable, while the independent variables included traumatic experiences during childbirth (IES-R subscale scores), postpartum depressive symptomatology (EPDS score), sleep disturbance (PSQI global score), and perceived social support (MSSS score). In addition, sociodemographic and obstetric variables, partner relationship satisfaction, and child-related factors such as child age were included as predictors to account for their potential influence on bonding outcomes. The assumptions for regression analysis were thoroughly assessed and met.

The Statistical Package for the Social Sciences (SPSS) version 29.0 (IBM Corporation in Armonk, NY, United States) was used to conduct all statistical analyses in this study.

## 4 Results

### 4.1 Sample size calculation

A preliminary calculation was performed to determine the appropriate sample size for the multiple linear regression analysis. This calculation assumed a medium effect size of *f^2^* = 0.15 (Faul et al., 2009), a power of 0.90, an *α* level of 0.05, and 20 predictors. The analysis indicated that a sample of at least 191 participants would be required to achieve adequate statistical power.

### 4.2 Sample characteristics

A total of 1,495 women completed the survey. The mean age of the participants was 32.35 years (SD = 4.34). Most participants were in a couple relationship (99.2%), and nearly half reported being completely happy in their relationship (47.8%). Detailed sociodemographic data are presented in Table 1.

**Table 1:** Sociodemographic characteristics of the study sample.

Sociodemographic variables		
Age		32.35 ± 4.34
Nationality	Italian	1,479 (98.9)
	Not Italian	16 (1.1)
Highest educational level	No educational qualification	1 (0.1)
	Primary school	3 (0.2)
	Middle school	76 (5.1)
	High school	647 (43.3)
	Bachelor’s degree	314 (21.0)
	Master’s degree	342 (22.9)
	Post-graduate degree	112 (7.5)
Employment	Unemployed	201 (13.4)
	Seeking first employment	19 (1.3)
	Student	33 (2.2)
	Armed forces	9 (0.6)
	Craftsman	30 (2.0)
	Employee	560 (37.5)
	Entrepreneur	33 (2.2)
	Freelancer	141 (9.4)
	Healthcare personnel	157 (10.5)
	Housekeeper	156 (10.4)
	Merchant	43 (2.9)
	School personnel	123 (8.2)
In a couple’s relationship	Yes	1,483 (99.2)
	No	12 (0.8)
Quality of the couple’s relationship	Not at all happy	13 (0.9)
	Somewhat unhappy	31 (2.1)
	Neither happy nor unhappy	195 (13.0)
	Somewhat happy	529 (35.4)
	Totally happy	715 (47.8)

Continuous variables are expressed as mean ± standard deviation and categorical variables as frequency (percentage).

The mean age of the infants was 6.39 months (SD = 3.48), with a mean birth weight of 3247.90 grams (SD = 484.07) and a mean Apgar score of 8.43 (SD = 2.30). The mode of delivery was heterogeneous, with the majority having spontaneous vaginal delivery (55.1%), followed by vaginal delivery with episiotomy (18.2%), unplanned cesarean section (16.9%), and planned cesarean section (9.8%). Primiparous women comprised 74.7% of participants. Approximately 29.0% of the participants reported risk factors during pregnancy, and 14.4% experienced hospitalization. Delivery complications were noted in 20.7% of the cases. In addition, 18.9% of the participants had a history of miscarriage, and 1.9% had a history of preterm delivery. Further details are provided in Table 2.

**Table 2:** Maternal and child characteristics in the study sample.

Variables		
Child age (months)		6.39 ± 3.48
Birth weight (g)		3247.90 ± 484.07
Apgar score		8.43 ± 2.30
Mode of birth	Spontaneous vaginal birth	824 (55.1)
	Vaginal birth with episiotomy	272 (18.2)
	Planned cesarean section	147 (9.8)
	Unplanned cesarean section	252 (16.9)
Parity	Primipara	1,117 (74.7)
	Multipara	378 (25.3)
Risk factors during pregnancy	Yes	433 (29.0)
	No	1,062 (71.0)
Hospitalizations during pregnancy	Yes	216 (14.4)
	No	1,279 (85.6)
Complications during delivery	Yes	310 (20.7)
	No	1,185 (79.3)
Previous miscarriages	Yes	283 (18.9)
	No	1,212 (81.1)
Previous preterm deliveries	Yes	28 (1.9)
	No	1,467 (98.1)

Continuous variables are expressed as mean ± standard deviation and categorical variables as frequency (percentage).

### 4.3 Descriptive statistics and correlations between questionnaire scores

Table 3 presents descriptive statistics and correlations of the study variables.

**Table 3:** Descriptive statistics and correlations for study variables.

Variable	M	SD	1	2	3	4	5	6	7	8
1. IES-R - Intrusion	13.50	5.33	-							
2. IES-R - Avoidment	7.10	5.43	0.680**	-						
3. IES-R - Hyperarousal	5.94	4.00	0.710**	0.750**	-					
4. IES-R - Total	26.53	14.67	0.900**	0.910**	0.890**	-				
5. MSSS	24.71	3.26	−0.072*	−0.068*	−0.075*	−0.080**	-			
6. EPDS	9.46	5.56	0.125**	0.110**	0.150**	0.135**	−0.480**	-		
7. MPAS	80.02	6.96	−0.050	−0.030	−0.060*	−0.070**	0.270**	−0.440**	-	
8. PSQI	6.09	3.50	0.500**	0.450**	0.520**	0.600**	−0.450**	0.620**	−0.550**	-

EPDS, Edinburgh Postnatal Depression Scale; IES-R, Impact of Event Scale – Revised; MPAS, Maternal Postnatal Attachment Scale; MSSS, Maternal Social Support Scale; PSQI, Pittsburgh Sleep Quality Index. *p < 0.05, **p < 0.01.

The MPAS had a mean score of 80.02 (SD = 6.96), indicating a generally good level of maternal bonding in our sample. The mean score for the IES-R Total was 26.53 (SD = 14.67), above the clinical cut-off of 24, indicating that participants, on average, reported a high level of childbirth-related post-traumatic stress symptoms. More specifically, 49.6% of the participants (*n* = 741) were classified as having significant post-traumatic stress symptoms. Subscale scores for Intrusion (*M* = 13.50, SD = 5.33), Avoidance (*M* = 7.10, SD = 5.43), and Hyperarousal (*M* = 5.94, SD = 4.00) reflected variability across symptom dimensions, with Intrusion being the most prevalent in the sample.

Although the mean EPDS score (*M* = 9.46; SD = 5.56) was below the cut-off of 12, indicating possible postpartum depression, the variability suggests that some participants may have experienced depressive symptoms. In fact, 32.2% of the participants (*n* = 481) passed the cut-off and were classified as having depressive symptoms.

The mean score of the PSQI was 6.09 (SD = 3.50), indicating that participants, on average, experienced sleep disturbances and suggesting potential sleep problems in the sample. Specifically, 50.7% of the sample (*n* = 758) reported sleep disturbances consistent with insomnia.

Finally, the MSSS had a mean score of 24.71 (SD = 3.26), indicating that most participants perceived adequate levels of social support. Notably, 57.9% (*n* = 866) of the sample reported adequate support, 36.9% (*n* = 551) reported medium support, and 5.2% (*n* = 78) were classified as having low social support.

Correlation analysis showed significant associations between several variables. The strongest correlations were found between PSQI (sleep quality) scores and total IES-R scores (*r* = 0.600, *p* < 0.01), indicating a significant link between poor sleep quality and childbirth-related post-traumatic stress symptoms. Likewise, the EPDS (depressive symptoms) scores demonstrated a strong positive correlation with the PSQI scores (*r* = 0.620, *p* < 0.01), suggesting that depressive symptoms are closely associated with sleep disturbances. Additionally, MPAS (mother-infant bonding) scores were negatively correlated with both PSQI (*r* = −0.550, *p* < 0.01) and EPDS (*r* = −0.440, *p* < 0.01) scores, indicating that poor sleep quality and higher depressive symptoms may be linked to weaker mother-infant bonding. In contrast, MSSS (social support) scores revealed a significant negative correlation with EPDS scores (*r* = −0.480, *p* < 0.01), emphasizing the protective effect of social support against depressive symptoms.

### 4.4 Differences in questionnaire scores based on child age

Table 4 summarizes the differences in the questionnaire scores based on the child’s age, emphasizing the statistical significance and effect sizes.

**Table 4:** MANOVA results with descriptive statistics.

	Child’s Age			*F* value	*p* value	Partial eta-squared
	0–4 months (*n* = 593)	5–8 months (*n* = 441)	9–12 months (*n* = 449)			
IES-R - Intrusion	15.50 ± 5.61	13.50 ± 5.28	11.50 ± 4.76	15.45	**< 0.001**	0.023
IES-R - avoidment	8.50 ± 5.43	7.00 ± 5.14	6.00 ± 4.62	10.85	**0.018**	0.010
IES-R - hyperarousal	7.50 ± 4.23	6.00 ± 3.92	5.00 ± 3.73	12.67	**0.001**	0.016
MSSS	23.50 ± 3.34	24.50 ± 3.18	25.50 ± 3.02	18.45	**< 0.001**	0.028
EPDS	11.50 ± 5.84	9.50 ± 5.42	8.50 ± 5.13	11.85	**< 0.001**	0.022
MPAS	78.50 ± 7.21	80.50 ± 6.76	81.50 ± 6.48	9.95	**0.001**	0.017
PSQI	7.50 ± 3.79	6.50 ± 3.45	5.50 ± 3.23	10.45	**0.002**	0.019

EPDS, Edinburgh Postnatal Depression Scale; IES-R, Impact of Event Scale – Revised; MPAS, Maternal Postnatal Attachment Scale; MSSS, Maternal Social Support Scale; PSQI, Pittsburgh Sleep Quality Index. Bold values indicate statistical significance at *p* < 0.05.

The MANOVA revealed significant differences between the child age groups (0–4 months, 5–8 months, and 9–12 months) for several variables. Childbirth-related post-traumatic stress symptoms, as measured by the IES-R subscales, were higher for mothers of younger children, with moderate effect sizes [e.g., IES-R Intrusion: *F*(2, 1,480) = 15.45, *p* < 0.001, η^2^ = 0.023]. The total IES-R score was excluded from the analysis to avoid multicollinearity problems, allowing a more focused examination of the individual subscales. EPDS scores also showed higher depressive symptoms in mothers of younger children, with a moderate effect size [F(2, 1,480) = 11.85, *p* < 0.001, η^2^ = 0.022]. In contrast, scores for social support (MSSS) and mother–child attachment (MPAS) improved with child age, with moderate effect sizes indicating significant associations [e.g., MSSS: F(2, 1,480) = 18.45, *p* < 0.001, η^2^ = 0.028; MPAS: F(2, 1,480) = 9.95, *p* = 0.001, η^2^ = 0.017]. Sleep quality (PSQI) also improved over time [F(2, 1,480) = 10.45, *p* = 0.002, η^2^ = 0.019].

### 4.5 Predictors of mother-infant bonding in the study sample

Multiple linear regression analysis was performed to examine the predictors of maternal postpartum bonding as measured by the total MPAS score. The predictor variables included age, birth weight, Apgar score, mode of delivery, presence of risk factors during pregnancy, hospitalization during pregnancy, delivery complications, history of spontaneous abortion, history of preterm delivery, presence of other children, partner relationship satisfaction, total PSQI, MSSS, and EPDS scores.

The regression model explained 31% of the variance in the total MPAS scores (*R^2^* = 0.31, adjusted *R^2^* = 0.30). ANOVA indicated that the regression model was a significant predictor of maternal postpartum bonding [*F*(20,1,440) = 26.978*, p* < 0.001].

The analysis identified several predictors that negatively influenced maternal–infant bonding. Maternal age was a significant negative predictor (*β* = −0.18, *p* < 0.001), with older mothers reporting lower bonding scores. Furthermore, unplanned cesarean delivery was associated with lower bonding (*β* = −0.08, *p* = 0.003), as were delivery complications (*β* = −0.05, *p* = 0.025) and the presence of risk factors during pregnancy (*β* = −0.06, *p* = 0.009). Psychological factors also emerged as significant predictors: higher scores on the Hyperarousal IES-R (*β* = −0.08, *p* = 0.038) and EPDS (*β* = −0.48, *p* < 0.001) were associated with weaker maternal bonding. In addition, sleep disturbance, as measured by the PSQI total score, was negatively associated with bonding (*β* = −0.07, *p* = 0.002).

In contrast, several predictors were positively associated with the MPAS scores. Mothers with other children reported higher MPAS scores (*β* = 0.07, *p* = 0.002). Higher levels of partner relationship satisfaction were significantly associated with better bonding (*β* = 0.12, *p* = 0.001). Finally, as measured by the total MSSS score, social support was also a significant positive predictor (*β* = 0.09, *p* = 0.005) of mother–child bonding. Table 5 reports all the statistical details of the regression model.

**Table 5:** Multiple linear regression predicting maternal bonding in the study sample.

Predictor	B	SE	*β*	*t*	*p*
(Constant)	89.90	2.49		36.11	**< 0.001**
Mother age	−0.30	0.04	−0.18	−6.67	**< 0.001**
Child’s age	0.02	0.06	0.008	0.36	0.721
Birth weight (kg)	−0.001	0.0	−0.02	−0.80	0.425
Apgar index	−0.005	0.07	−0.002	−0.07	0.946
Mode of delivery (Unplanned Cesarean Section)	−1.50	0.50	−0.08	−3.00	**0.003**
Mode of delivery (Planned Cesarean Section)	0.78	0.50	0.04	1.56	0.119
Mode of delivery (Vaginal with Episiotomy)	0.67	0.45	0.04	1.49	0.137
Risk factors during pregnancy	−0.98	0.37	−0.06	−2.62	**0.009**
Hospitalization during pregnancy	0.35	0.47	0.02	0.74	0.459
Complications during delivery	−1.00	0.44	−0.05	−2.26	**0.025**
History of spontaneous abortions	0.39	0.42	0.02	0.92	0.356
History of preterm deliveries	0.94	1.22	0.02	0.77	0.444
Presence of other children	1.20	0.39	0.07	3.06	**0.002**
Happiness in partner relationship	0.80	0.20	0.12	4.00	**0.001**
IES-R intrusion	−0.05	0.03	−0.05	−1.47	**0.014**
IES-R avoidment	−0.02	0.04	−0.02	−0.51	0.609
IES-R hyperarousal	−0.100	0.05	−0.08	−2.08	**0.038**
PSQI	−0.10	0.03	−0.07	−3.12	**0.002**
MSSS	0.20	0.07	0.09	2.86	**0.005**
EPDS	−0.60	0.03	−0.48	−17.64	**< 0.001**

R^2^ = 0.312, Adjusted R^2^ = 0.302. B, unstandardized coefficient; SE, standard error; β, standardized coefficient; EPDS, Edinburgh Postnatal Depression Scale; IES-R, Impact of Event Scale – Revised; MPAS, Maternal Postnatal Attachment Scale; MSSS, Maternal Social Support Scale; PSQI, Pittsburgh Sleep Quality Index. The reference category for the mode of delivery is spontaneous vaginal birth. Values in bold indicate statistical significance at *p* < 0.05.

## 5 Discussion

This study aimed to explore the key factors affecting mother-infant bonding during the first year of a child’s life by focusing on risk and protective variables. It specifically examined how maternal mental health, birth trauma, sleep disturbances, perceived social support, and partner relationship satisfaction shape the quality of early mother-infant bonding. By analyzing the predictors of bonding quality and examining variations based on the child’s age, this study seeks to offer valuable insights for interventions promoting healthy mother–child bonding from the early postpartum period, ultimately supporting healthy and balanced child development.

The descriptive statistics revealed remarkable patterns in maternal psychological well-being and bonding experiences. The overall level of maternal bonding in the study sample was good. However, a significant percentage of participants scored above the clinical threshold on the IES-R and PSQI (49.6 and 50.7%, respectively), indicating a significant presence of post-traumatic stress symptoms related to childbirth and sleep disturbance, respectively.

Notably, the observed prevalence of postpartum PTSD symptoms in our sample (49.6%) exceeds the range typically reported in the literature, which varies from 1 to 30% (Ayers et al., 2006; Grekin and O'Hara, 2014). Several factors may explain this discrepancy. First, this study was based on self-reported symptoms measured by the IES-R, a validated screening tool designed to assess symptom severity rather than provide a clinical diagnosis. As such, the tool may have identified a broader range of psychological reactions, potentially leading to an overestimation of clinically significant PTSD. Second, the online recruitment strategy through social media and parent forums may have introduced a self-selection bias, attracting mothers who were experiencing severe psychological distress or actively seeking support. Third, the timing of the assessment, which spanned the entire first year postpartum, may have captured symptoms that emerged or persisted over time, particularly among mothers without adequate psychosocial support.

Despite this high prevalence in our sample, these findings remain consistent with previous research that points out that a substantial proportion of women experience symptoms of post-traumatic stress after childbirth, which can negatively affect the quality of maternal bonding (Ayers et al., 2006; Grekin and O'Hara, 2014). Childbirth-related PTSD has been linked to maternal emotional detachment and decreased sensitivity in nurturing behaviors, which are critical for establishing a secure attachment between the mother and child (Dekel et al., 2017; Dekel et al., 2019). Symptoms, such as hypervigilance, irritability, and intrusive memories, can disrupt a mother’s capacity to participate in nurturing interactions, affecting the child’s emotional and social development (Beck et al., 2011).

Sleep disturbances are common during the postpartum period and are closely linked to reduced maternal emotional sensitivity and availability (Tikotzky, 2016). Poor sleep quality can worsen psychological issues, such as anxiety and depression, creating a negative cycle that further affects the bonding experience with the child (Hiscock et al., 2014; Hairston et al., 2016). Extended periods of sleep disturbance are also associated with a higher risk of maternal fatigue, emotional instability, and increased irritability, all of which can impede the formation of a secure bond between mother and infant (King et al., 2020).

Although the mean EPDS scores were below the clinical threshold for postpartum depression, there was individual variability in the depressive symptoms. This finding is consistent with studies suggesting that subclinical depressive symptoms can impair maternal functioning and bonding (Beck, 2001; Tietz et al., 2014). Even mild depressive symptoms can reduce a mother’s emotional readiness, ability to accurately interpret infant cues, and confidence in caregiving (Humphreys et al., 2018). Therefore, research suggests that subclinical depressive symptoms are associated with avoidant or disengaged caregiving behaviors, which can negatively affect an infant’s emotional security and attachment patterns (Field, 2010).

The analysis of age-related differences in psychological outcomes has revealed that mothers with younger infants (0–4 months) face significant challenges, reporting higher levels of post-traumatic stress and depressive symptoms than those with older infants (5–8 months and 9–12 months). This finding aligns with existing evidence indicating that the early postpartum period is a time of heightened vulnerability due to hormonal changes, sleep deprivation, and adjustment to new caregiving responsibilities (Yim et al., 2015; Kendig et al., 2017). New mothers often navigate a steep learning curve in meeting their babies’ needs while also recovering physically from childbirth, which can intensify psychological distress (Saharoy et al., 2023). On a positive note, our study found that maternal social support and the bond between mothers and children tend to improve as children grow older. This result likely indicates that mothers gradually adapt to their caregiving roles and build stronger social networks over time (Dennis and Letourneau, 2007; Leahy-Warren et al., 2012). As children grow, they usually establish more predictable sleep and feeding routines, which ease the burden on mothers and allow for better emotional and physical recovery (Hiscock et al., 2014). This transition often leads to increased confidence in caregiving as mothers become more skillful in understanding and responding to their children’s needs, thus enhancing their maternal self-efficacy (Bandura, 1997; Leahy-Warren and McCarthy, 2011). In addition, higher levels of perceived social support play a protective role, buffering stress and promoting resilience in the face of postpartum challenges such as breastfeeding (Schmied et al., 2011; Negron et al., 2013). Emotional support from partners and family members and practical assistance allow mothers to devote more time and energy to bonding with their newborns (Stoodley et al., 2025). Based on our findings, sleep quality also appears to improve as the child ages, further supporting the trend that better sleep is associated with greater maternal well-being and caregiving ability. Studies have shown that enhanced maternal sleep is associated with reduced depressive symptoms, greater emotional readiness, and better mother-infant interactions (Tikotzky, 2016; Mindell and Williamson, 2018).

The main goal of this study was to identify the factors that predicted mother–child bonding in our sample, considering various aspects related to the mother, child, and their experiences during pregnancy and childbirth. Specifically, the regression analysis sheds light on some of the variables that influence mother–child bonding. Among the factors analyzed, depressive symptoms stood out as the most significant negative predictor, with a beta coefficient of −0.48, highlighting the substantial effect of maternal mental health on the quality of the relationship with the child. This finding aligns with a wealth of research linking postpartum depression to reduced maternal sensitivity, diminished emotional availability, and challenges in developing secure attachments (Figueiredo et al., 2009; Slade et al., 2020). Additionally, maternal age was identified as another significant negative predictor of mother–child bonding (*β* = −0.18), which is in contrast to existing studies suggesting that younger mothers often exhibit lower caregiving skills, making it harder for them to bond effectively with their infants (De Falco et al., 2014; Ahsan et al., 2023). One possible reason for this unexpected result could be that older mothers experience heightened stress and physical challenges related to aging, which may hinder their ability to fully engage in caregiving.

Furthermore, older mothers may have higher expectations and increased anxiety about parenting, potentially leading to a perceived or actual decline in the quality of their bonding. It is also possible that older mothers face more significant work or career obligations, limiting the time and energy they can dedicate to building strong connections with their infants. Additionally, older mothers may be at a greater risk of experiencing health complications during pregnancy and postpartum, which could impact their overall well-being and capacity to bond effectively with their children.

Traumatic experiences related to childbirth, particularly hyperarousal symptoms, as measured by the IES-R (*β* = −0.08) and sleep disturbance (PSQI, *β* = −0.07), were also found to be negative predictors of maternal bonding quality. In this regard, research has shown that maternal PTSD symptoms, particularly hyperarousal, are prospectively associated with poorer mother-infant bonding. One study reported that maternal PTSD symptoms at 1 month postpartum hurt attachment at 3 months, with post-traumatic symptoms mediating this relationship, specifically intrusion and hyperarousal symptoms (Stuijfzand et al., 2020). The mechanisms underlying this association may be related to the effect of hyperarousal on emotional readiness and maternal sensitivity. When mothers experience hyperarousal, it can result in heightened irritability and emotional instability, which can hinder their ability to respond sensitively to infants’ needs. Furthermore, sleep disturbances linked to hyperarousal can worsen fatigue, making it even more challenging for mothers to be responsive (Di Blasio et al., 2018).

Interestingly, unplanned cesarean deliveries (*β* = −0.08), the presence of risk factors during pregnancy (*β* = −0.06), and complications during delivery (*β* = −0.05) further contributed to poorer bonding quality among women in our sample, consistent with research linking adverse obstetric experiences to feelings of loss of control and reduced confidence in the caregiving role (Rowlands and Redshaw, 2012; Sommerlad et al., 2021).

The regression model also allowed us to identify variables that appeared to act as protective factors in relation to the quality of mother–child bonding. Specifically, partner relationship satisfaction showed a strong positive association with bonding (*β* = 0.12), underscoring the critical role of emotional and practical partner support in reducing postpartum stress and enhancing maternal bonding (La Rosa et al., 2024). The perceived social support score (MSSS, *β* = 0.09) was also a significant positive predictor, further emphasizing the importance of a strong support network in promoting positive outcomes, as perceived social support has been shown to alleviate postpartum depression and encourage healthier mother-infant interactions (Dennis and Letourneau, 2007; Vaezi et al., 2019; De Sousa Machado et al., 2020). Interestingly, having other children was associated with stronger bonding scores (*β* = 0.07), suggesting that prior parenting experience strengthens trust and reduces caregiving anxiety.

### 5.1 Study limitations

Although this study presents valuable findings, it is important to recognize several limitations. First, the cross-sectional design restricts our ability to determine causal relationships among the variables observed. Longitudinal studies are necessary to confirm the directionality of these relationships and to investigate how maternal psychological well-being, social support, and sleep quality affect the mother-infant relationship over time. Second, relying on self-report measures such as the IES-R, EPDS, and PSQI may introduce bias, as participants might under or over-report their symptoms due to recall or social desirability issues. Future research should incorporate objective measures, such as actigraphy, to assess sleep quality, and structured clinical interviews to evaluate depressive and post-traumatic stress symptoms to validate self-reported outcomes.

Third, recruiting participants through social media and online platforms poses challenges regarding sample representativeness. Although this method enabled the inclusion of a large sample, it may have led to a selection bias, which could limit the generalizability of the findings to broader or more diverse populations. Future studies should strive to recruit participants from varied and representative backgrounds, including mothers from different socioeconomic and cultural contexts. Fourth, this study did not specifically examine the effects of maternal anxiety symptoms, which often co-occur with postpartum depression and have been shown to independently affect maternal sensitivity and bonding (Fallon et al., 2021). Therefore, it is advisable to include anxiety as a variable in future research to enhance our understanding of the psychological factors that influence mother-infant bonding. Lastly, while the study identified several significant predictors of maternal–infant bonding, the regression model accounted for only a portion of the variance in the bonding scores. This finding indicates that other unmeasured factors may also play a crucial role. Future studies should consider other variables, such as breastfeeding practices, infant temperament, and cultural influences, to provide a more detailed perspective on the determinants of maternal bonding. In addition, investigating the interaction between these factors and the identified predictors, such as depressive symptoms and social support, could improve our understanding of the complex dynamics that shape the mother-infant relationship from the earliest months of a child’s life.

## 6 Conclusion

The results of this study have important implications for developmental psychologists and clinicians, particularly about the early bonds between mothers and infants. The quality of these early relationships is critical in shaping the child’s future development, and the importance of secure bonds formed early in life cannot be overstated. These secure bonds lay the foundation for healthy emotional and social development and influence future interpersonal relationships, emotion regulation, and psychological resilience throughout life. Therefore, it is essential to implement preventive measures early in a child’s life to address the risks associated with insecure attachment.

To this end, early screening for maternal mental health conditions, particularly postpartum depression, post-traumatic stress symptoms, and sleep disorders, should be systematically integrated into prenatal and postnatal care. These screenings should be sensitive not only to clinically significant disorders but also to subclinical symptomatology that may still impair emotional readiness and maternal bonding. Early identification of mothers at risk allows for early intervention to reduce distress and promote more responsive mother-infant interactions. Even mothers who do not meet the diagnostic threshold for mental disorders can benefit from brief, evidence-based interventions that focus on emotional support, psychoeducation, or behavioral strategies.

Mental health services should be embedded in multidisciplinary perinatal care teams and made available through public health systems to ensure equitable access. Support may include individual therapy, group counseling, or digital mental health resources adapted to the postpartum period. In parallel, improving maternal sleep quality is critical, as sleep disturbances are common in the first few months and can significantly affect the mother’s mood and ability to bond. Interventions such as sleep hygiene education, cognitive-behavioral approaches to insomnia, relaxation techniques, and structured sleep support programs have shown promising results and should be incorporated into standard postpartum care.

In addition to addressing mothers’ psychological and physiological needs, it is equally important to strengthen social support networks, particularly the role of partners. Evidence shows that when fathers are actively involved in caregiving and provide emotional support, maternal stress is reduced, and bonding with the newborn is enhanced. Health policies should promote paternal involvement through educational initiatives, couple-based interventions, and flexible parental leave policies that encourage shared responsibility in the early stages of caregiving. Parenting workshops and peer support groups can also enrich mothers’ social networks, reduce isolation, and increase perceived competence and emotional well-being.

These findings highlight the need for a comprehensive, family-centered approach to postnatal care that integrates psychological assessment, sleep support, and relational interventions into routine practice. Such an approach not only benefits mothers and infants individually but also contributes to broader public health goals by supporting early relationship health and preventing the long-term consequences of compromised attachment.

In conclusion, this study highlights the complex interaction of psychological, physiological, and relational factors that influence maternal bonding in the postpartum period and the importance of a multidimensional and preventive approach to perinatal care. Future research should focus on longitudinal and intervention studies to explore the evolution of these factors over time and to evaluate the effectiveness of targeted strategies in different populations and cultural contexts. A deeper understanding of these dynamics can inform policy development, improve clinical practice, and ultimately support healthier parent–child relationships during this critical developmental period.

## Data Availability

The dataset presented in this article is not readily available due to participant privacy concerns. Requests for access to the dataset should be directed to the corresponding author.
